# Simulated gestation: The social and ethical implications of in vivo fertilisation technology

**DOI:** 10.1111/bioe.13416

**Published:** 2025-04-16

**Authors:** Ji‐Young Lee, Adrian Villalba, Natalia Fernández‐Jimeno

**Affiliations:** ^1^ Department of Public Health University of Copenhagen Copenhagen Denmark; ^2^ Institut Cochin, CNRS, Inserm Université Paris Cité Paris France; ^3^ Department of Philosophy II University of Granada Granada Spain; ^4^ Department of Science, Technology and Society, Institute of Philosophy Spanish National Research Council Spain

**Keywords:** assisted reproduction, gestation, INVOcell, pregnancy, simulated gestation

## Abstract

INVOcell is an in vivo fertilisation device marketed as an alternative to in vitro fertilisation treatment. In this paper, we explore the ethical implications that arise when this device is framed as a type or process of ‘gestation’. We anticipate several effects that may be of ethical interest: marketing in vivo fertilisation as being comparable to traditional gestation may be misleading and even harmful to its users, but on the other hand, it captures a potential need to acknowledge and be more inclusive of those who wish to identify diverse reproductive experiences as *gestative* experiences. In light of these insights, we articulate the concept of ‘simulated gestation’ as a novel framework to explain, and potentially accommodate, the diverse ways in which assisted reproductive technologies are shaping people's ideas about—and desires for—gestation.

## 1 INTRODUCTION

‘INVOcell’ is the first FDA‐approved medical device for the treatment of infertility via intravaginal culture (IVC).^1^ The device in question is loaded with eggs and sperm and then inserted into a woman's vagina for *in vivo fertilisation*. This means that fertilisation and the initial stages of embryonic development will occur within the patient's body, rather than in a laboratory setting, as would have been the case for *in vitro fertilisation* (IVF).

The preparatory phases of the INVOcell technique mirror those of IVF. Initially, ovarian stimulation is conducted to foster the development of a moderate number of ovarian follicles in the recipient. Subsequently, follicular puncture is scheduled to procure the oocytes from the patient, followed by the collection and preparation of the sperm. Contrary to IVF, however, in INVOcell, the eggs and sperm are loaded onto the specialised culture device. Up to seven egg cells may be introduced to the INVOcell device. Finally, a gynaecologist places the INVOcell device into the patient's vagina, where it remains for a span of 3 days, facilitating fertilisation and the first embryonic divisions. After this period, the gynaecologist will remove the device from the patient. Once the device is removed, it is assessed for the number of viable embryos that have resulted from the process, and the selection of those to be transferred to the recipient's uterus for implantation.^2^ Surplus embryos may be cryopreserved for subsequent transfers.

As such, this procedure circumvents the incubation of embryos in the laboratory. The main advantage of the INVOcell protocol is marketed as follows: “It enables women to be more involved in the assisted reproduction process and can be an option when conventional IVF is not accepted for ethical, religious, or cultural reasons.”^3^ By *enabling patients to have greater involvement in the assisted reproduction process*, the use of INVOcell has been claimed to allow “two‐mother” gestation in the case of lesbian couples. The reason it is dubbed ‘two‐mother’ gestation is because one partner carries the INVOcell device during the first 3 days (in vivo fertilisation), whereas the other partner will then receive the resulting embryo for the gestational period until birth. Hence, both partners are respectively able to participate in this assisted reproductive process with their bodies. This duplication of gestational motherhood is how it is framed both in the scientific literature (‘Vaginal culture for IVF allows two mothers to carry the same pregnancy’),^4^ in the media (*How Did Two Mothers Both Carry the Same Child?* 5 or *Europe's First Pregnancy Shared By Two Women* 6), among many other cases, and indeed by the company marketing the product.

In 2018, Ashleigh and Bliss, a couple from the United States, were the first lesbian couple to successfully carry a full pregnancy in which the embryo was fertilised in the INVOcell device. By using INVOcell, Bliss underwent the initial stages of assisted reproduction by having her eggs fertilised with donor sperm and then frozen after 5 days of incubation. Meanwhile, Ashleigh proceeded with hormonal treatments in preparation for the subsequent embryo transfer, which was successfully implanted and carried on with the pending stages of pregnancy. In 2023, INVOcell was used for the first time in Europe by a lesbian couple.

In our view, this in vivo fertilisation device presents a very current and rather curious example of a way that assisted reproductive technologies (ART) serve to reinforce, and at the same time to redraw, the meanings and boundaries of gestation and motherhood. We therefore offer several points for critical reflection in our paper. In Section 2, we consider INVOcell as a dynamic socio‐technical system that targets certain ART‐seeking groups, especially lesbian couples. We contend that INVOcell invokes (problematic) naturalistic and essentialist narratives about motherhood to legitimise INVOcell as a type or process of gestation. In Section 3, we consider the potential ethical impact of framing devices like INVOcell as a form of gestation, identifying not only situations in which it may pose harm but also those in which it could promote inclusivity. This inquiry leads us to delineate the concept of *simulated gestation*, with the objective of evaluating how emerging ART can reshape societal perceptions about access to gestation by offering novel or alternative routes into gestational experiences. Altogether, our article presents an anticipatory exploration of the ethical concerns that may arise from the use of IVC devices and discusses its ramifications for traditional conceptions of gestation.

## 2 INVOCELL: NARRATIVISING IN VIVO FERTILISATION AS EMBODIED GESTATION

According to the Oxford English dictionary^7^, gestation *is the action or process of carrying the young; the condition of being carried in the womb during the period between conception and birth*. As a more clinical term, the Royal College of Obstetricians and Gynaecologists refers to gestation as *the time between conception and birth, when the fetus grows and develops inside the mother's womb*. Both conceptions pay strong attention to the term *womb*, signifying the role of the uterus as an essential component of pregnancy. This is the traditional understanding of gestation.

Since the birth of Louise Brown in 1978, significant advancements in reproductive technologies have reshaped the traditional understanding of gestation.^8^ Thanks to IVF, it is now evident that a portion of the time between conception and birth can partially occur outside the maternal body, within the controlled environment of a laboratory dish. If IVF is used, the term ‘gestation’ denotes the period from embryo transfer until birth, rather than applied to the initial stages of fertilisation and culturing of embryos in the laboratory, typically lasting around 5 days. Thus, in the case of IVF, the process of gestation is considered to be delayed until the embryo transfer takes place. Despite this technical difference, however, successful conception via embryo transfer after IVF would more or less replicate the embodied pregnancy experienced by those who conceive naturally. Thus, IVF users can achieve gestation (traditionally understood) for the rest of the gestational period subsequent to embryo transfer.

INVOcell pushes these boundaries even further by claiming, as we explain later, that in vivo fertilisation itself is sufficient to replicate traditional gestation. In order to address the normative potential that INVOcell has to both reinforce and reshape its users' understanding of, and relationship to, gestation, we must first critically observe the ways in which the entity producing and marketing the device imputes in vivo fertilisation *as* a process of legitimate gestation. Let us now investigate how the INVOcell device functions within the broader system of ART to better understand the potential and relevance of this technology.

First, it can plausibly be claimed that ART is a system that has evolved a great deal since the invention of IVF and has become increasingly complex. IVF has created around it a whole series of accessory and complementary technologies such as sperm selection (MACS), intracytospermic injection (ICSI), gamete donation, gamete and embryo cryopreservation, just to mention some of the best known ones. In the meantime, the following instruments have also been developed for oocyte retrieval: needles and developments in laparoscopy, adjusted hormone doses, or other artefacts to improve laboratory procedures or the preservation of embryos in hatcheries.^9^ Also, as sociologist Sarah Franklin has described, IVF can be understood as a ‘platform technology’ that has supported other technological developments, such as Preimplantation Genetic Diagnosis (PGD) or stem cell research.^10^ But ART is also a heterogeneous system, as it is not only composed of technologies and artefacts but also human actors that make the system work, such as gynaecologists, nurses, embryologists, sales staff, managers, researchers, and so forth. There are also scientific societies (e.g., European Society of Human Reproduction and Embryology [ESHRE]), companies and clinics, and assisted reproduction laws and regulations. It is important to note that in this complex system, the social elements are as important as the artefacts. The social dimension is made up of agents who delineate the possible trajectories through which these technologies evolve. These actors induce or inhibit the ways in which technologies can be used, inscribing their values in the technologies.^11^ They imagine the ways in which these technologies might be used, constructing collective narratives and social meanings that change over time and according to various contexts.^12^

It is relevant to note that as large dynamic socio‐technical systems such as ART mature and acquire momentum, they try to control their environment.^13^ To do so, they have to make adaptations. For example, ART has always targeted heterosexual couples, but in recent years, it has had to accommodate a new type of user: lesbian couples who seek ART, without necessarily having issues related to medical infertility. To cope with this type of user, the ART system offered these couples donor insemination. Over time, it has developed novel technologies (such as Reception of Oocytes from Partner (ROPA) or INVOcell) and discourses about them as a way of making these products attractive to these users. This broadens the market and creates new marketing needs that previously did not exist because lesbian couples did not value biological links as much in the development of kinship ties.^14^ It is important to note, however, that since the routinisation of ART, the system and its builders (Robert Edwards, Jean Purdy and Patrick Steptoe) have valued genetic links in the creation of kinship relations and have sought to preserve them through various technologies and discourses.^15^ We hypothesise that INVOcell contributes to the maintenance of this system, as we explain below.

First, INVOcell endeavours to entice those who object to the use of IVF for ethical, social or religious reasons. On the product's webpage, INVOcell is positively marketed as an ‘an innovative approach where a woman's body acts as a natural incubator for fertilisation’.^16^ It further claims that INVOcell, in *contrast* to IVF, ‘keeps women closely connected to the experience’, presumably to the experience of conception‐to‐gestation. Such appeals exploit the possibility that some ART users may have pre‐existing objections or reservations about IVF. For instance, some religious individuals reject extracorporeal fertilisation^17^ as a practice associated with IVF. In this manner, INVOcell appeals to an audience^18^ that may be sceptical of ART to some degree, by reassuring potential users of the ‘natural’ nature of the device and the active role that users can gain from employing the device. On their website, *INVO Bioscience* addresses potential users in this way: ‘INVOcell gives you the opportunity to be an integral part of the process right from the very beginning’.^19^ The narrative constructed by this company places the users at the centre of the treatment, instilling in them a sense of control and autonomy over the process.

However, these claims about the value of ‘natural’ in vivo fertilisation relative to in vitro fertilisation are made without further explanation or argument, which, in our view, makes INVOcell's marketing campaign committed to a type of ‘naturalistic fallacy’, in which whatever is claimed to be ‘natural’ is erroneously treated as tantamount to what is morally ‘good’.^20^ When this ‘fallacy’ is wielded in concert with (oppressive) social norms about motherhood to discredit certain biotechnologies or interventions, the potential harms are quite clear. For example, promoting the view that a natural, vaginal birth is better than medicalised births like C‐sections might actually reinforce a negative birth experience for those who believe that they have failed to achieve the ‘good’ mode of birth.^21^ In any case, bioethicists have cautioned against assuming that concepts of naturalness/unnaturalness are a reliable way to intuit the moral acceptability of biomedical technologies.^22^ The false dichotomisation of IVF as unnatural and therefore ‘bad’, versus INVOcell as natural and therefore ‘good’, is a normative flaw with the INVOcell campaign.

Second, INVOcell claims to transform the process of assisted reproduction into an ‘experience more intimate and personal’, allegedly enabling the patient to assume a leading, *causal* role in the fertilisation process. It is said^23^ that they will facilitate the formation of a bond between the future parents and their unborn child from the earliest stages of pregnancy. These ideas are supported by framing the *in vivo* approach as a most relevant and desirable method for aspiring mothers, highlighting the process of conception and first embryonic development as crucially taking place in the woman's body. As an illustration, consider the following text, which is addressed to potential users on the INVO Bioscience company website^24^:

At INVO Bioscience our approach to fertility care is simple—it begins with you. Through *its in vivo* approach, INVOcell® offers an intimate experience with impressive success rates […] INVOcell allows fertilisation and early embryo development to take place ***within the woman's body.*** It creates efficiencies throughout the whole process, making it easier for you.

Here, they frame INVOcell by exploiting the common idea that *in utero* fertilisation brings women closer to gestational motherhood, the latter of which is commonly considered a highly valuable—if not the *sole*—basis for maternal connection with the would‐be child.^25^ INVOcell thus plays on biomedical metaphors that depict the female body as a ‘container’ for nurturing a developing foetus,^26^ or perhaps more pejoratively on the stereotypical notion that women can only become true mothers through pregnancy and childbirth.

This depiction is worrying, however, because there are several reasons to think that in vivo fertilisation and the concept of female bodies as ‘incubators’ are highly misleading, if not outright oppressive.^27^ The initial flaw in this part of the narrative lies in its idealisation of the female body's role in the formation of motherhood, relying only on the aspect of biological embodiment as relevant. The notion that gestation is the ideal pathway to attain motherhood may encourage women unable to carry pregnancies to believe that the experience of gestation is necessary to earn full motherhood.^28^ This is in line with the genetic essentialism that ‘define parental and offspring roles largely though not exclusively in genetic or biologic terms’,^29^ which thereby frames the use of assisted reproductive technologies as a preferable path to family‐making relative to other options like adoption. Such beliefs serve to diminish the legitimacy of diverse and ‘alternative’ pathways into motherhood. For example, mothers who commissioned a surrogate to bring their child into the world never gestated themselves, and yet, this does not make them any less a ‘mother’ to the resulting child. This similarly applies to mothers who adopted their children: just because they did not gestate and give birth to the child does not mean that they are not mothers.

Additionally, we want to emphasise here that it is not biologically necessary for the INVOcell device to be situated inside a woman's body for the fertilisation process to occur. Thus, the company appears to actively commit a scientific falsity by claiming that intravaginal fertilisation via INVOcell is constitutive of gestation traditionally understood. With INVOcell, it is the device itself that fosters the fertilisation process. INVOcell's closed system does not, therefore, directly engage with the woman's body, in contrast to natural pre‐implantation development, where the maternal organism *is* an active participant in the developmental process of an embryo. Moreover, the entire process of embryo fertilisation and early development can be conducted in a laboratory without the need for a uterus or this specific device, because of IVF. When IVF is used for implantation, as we have already mentioned, it is widely accepted that ‘gestation’ occurs after the embryo transfer, not before. Thus, promoting the notion that the female body serves as a causally active incubator for the fertilisation‐to‐gestation process in INVOcell, just because the fertilisation device is physically placed in the user's vagina rather than kept in a lab, is false to begin with.

Finally, both the INVO Bioscience and INVOcell Life Begins Within websites display photographs of heterosexual couples of different ethnicities, as well as female couples. They are in effect summoning narratives about inclusivity and diversity to frame their product as a positive experience for all families. This approach has been used by Juaneda Hospitales, which claims to be the first clinic to utilise this method on female couples. Juaneda Hospitales^30^ asserts that ‘this procedure confers significant emotional value, as both women share the experience of gestating the embryo’.

The possibility of offering lesbian couples a shared experience of pregnancy is presented as a principal advantage of the system at hand. Other than INVOcell, The ROPA method has been described as a means of creating a discourse of ‘shared motherhood’ insofar as both women contribute part of their genetic information.^31^ The INVOcell method also purports to offer a shared gestational experience by giving users a sense of equal involvement and participation in the process of fertilisation‐to‐gestation. In this way, the options for lesbian couples as consumers are increased by offering them not only a way of bonding on a genetic and epigenetic level, as was the case with the ROPA method, but also by bonding through a shared experience of gestation. One partner will experience gestation by carrying the baby in her uterus, while the other will act as a so‐called ‘incubator’ to the INVOcell device during the first cell divisions and the formation of the embryo. In so doing, the non‐gestating lesbian partner is asked to envisage that she has assumed a role in the care and nurturing of the embryo.

An ambivalent moral consequence of encouraging this narrativisation of shared gestation for lesbian couples is the fact that this reinforces the idea that ‘motherhood’ can and should be reduced to *gestation*. By suggesting that the female body is the ideal facilitator of embryo fertilisation, it implies that at least ‘incubating’ an embryo is sufficient to attain a primary objective of motherhood: namely, to participate in the gestational process. This undervalues the significance of *social* maternal roles while placing undue emphasis on the biological aspects of reproduction. As with other reproductive technologies, there is a paradox in that the formation of the family is understood and valued as a biological fact, while at the same time, the cultural practices that construct it are omitted.^32^ Consequently, the maternal–filial bonds, which are de facto a social construction resulting from the attachment that develops in the mother–child social relationship, are made secondary to the purported value of biological bonds that are narratively and performatively reconstructed in the theatre of imitating nature with the assistance of INVOcell. Thus, in patriarchal social settings that gender the value of biological bonds for the formation of kinship over and above social relationships, the system is able to mobilise essentialist social norms—and even sketchy scientific claims—about coming into motherhood as a strategy to lure in service users who are motivated by desires to fulfil such motherhood norms.

Ultimately, our view is that INVOcell seeks to capture and compete for ART users by appealing to several audiences entangled in reproductive care‐seeking: individuals who are hesitant about IVF, those who wish to exercise embodied control over their intracorporeal and ‘natural’ fertilisation, and lesbian couples who hope to share more equally in their experiences of coming into motherhood. As we have commented on throughout our analysis, INVOcell banks on the social force of pre‐existing stereotypes about the value of gestational motherhood as a way to renew naturalistic and essentialist ideas about what constitutes preferable forms of embryo‐making in the case that women's bodies, rather than laboratories, can be used after all. Consequently, INVOcell is presented as a device that mimics the ‘natural’ process of fertilisation if utilised inside the woman's body as the vessel and guardian of the process. It is these forceful social claims and expectations of motherhood that allow the aforementioned actors to be drawn into the system, as we have seen in the case of lesbian couples. To the extent that potential users already espouse bio‐normative values, they may be more readily attracted to the ART system.

## 3 INVOCELL AND THE CONCEPT OF SIMULATED GESTATION

Given the various holes and flaws with the INVOcell campaign observed in the previous section, one might question why INVOcell has been so convincing for the lesbian couples whom we mentioned in the beginning of our article. Our perspective is that, despite INVOcell not actually fulfilling the conditions of gestation *traditionally* understood, which is what it promised to provide originally, it nevertheless appears to successfully satisfy phenomenological desires for traditional gestation by tapping into the users’ pre‐existing gendered narratives surrounding the journey into motherhood. For example, a woman who opts for a surrogate to carry her child may be drawn to INVOcell as a means to participate in the process of conception‐to‐gestation first‐hand, if she believes that intravaginal fertilisation is sufficient to count as gestation, or would be a satisfactory alternative to full gestation, rather than be left to feel that gestation must be entirely outsourced from her body. Therefore, even though INVOcell is technically just an in vivo fertilisation device that does not causally require the involvement of a female body to carry out its function, it may nevertheless fulfil compensatory desires for gestation in cases where traditional gestation cannot be achieved—such as when two women in a lesbian partnership both desire to gestate the same foetus. These are just a few examples of ways in which INVOcell could potentially influence individuals to reinscribe traditional understandings of ‘gestation’ in ways that will match their own experiential needs and desires.

Further qualitative research would be necessary to fully understand the potential narrative trajectories of gestation enabled by assisted reproductive devices like INVOcell. Taking this into account, it is also worth pointing out the potentially positive and inclusivity‐promoting features of the narrative that INVOcell enables ‘gestation’. While we would still maintain that the overall INVOcell protocol contains ethical issues and fails to satisfy the biotechnical conditions of gestation discussed in the previous section, the association made between gestation and INVOcell as an in vivo fertilisation device, in our view, prompts further reflection on the very meaning of ‘gestation’ used outside of a strictly biomedical context. We will thereafter outline a concept that we call ‘simulated gestation’ as a way to give importance to subjective identifications with gestation and to capture the notion that meanings attributed to ‘gestation’ are dynamic and continuing to change over time.

It is worth noting, first, that reproductive medicine has already shifted modalities of conception and gestation, and more generally what it means to engage in family‐making. ‘Traditional’ or ‘natural’ reproduction implies that the conception‐to‐gestation process occurs entirely in vivo, since intercourse is sufficient for implantation and pregnancy. In this instance, furthermore, the gestator is necessarily a genetic parent, and likely also the social and legal parent. This explains why the principle of *mater semper certa est* 33 is a legal norm in many countries, like in Spain, meaning that the person who gives birth to the child is automatically the legal mother of the child.

In a world with IVF, however, the principle of *mater semper certa est* is challenged due to the social changes that IVF have brought about with regard to the meaning of motherhood and parenthood. The conception‐to‐gestation process can be manipulated with IVF: because fertilisation of eggs and sperm can be done in vitro, it is also possible to conduct embryo transfer for agents who are not necessarily the genetic or intended parent. This is the norm with *gestational surrogacy*, in which someone other than the genetic/intended mother acts as a gestational carrier. If all goes well, the gestational carrier would be expected to carry the pregnancy but to relinquish legal and social parenthood after birth. Hence, in this case, the principle of *mater semper certa est* is insufficient to describe the ways in which people might renegotiate the boundaries of what it means to be a ‘parent’ or ‘mother’.

The idea that gestation is *not* necessary to become a parent/mother (as is the case with adoptive parents, or parents who use surrogates) can therefore be legitimised in the context of a world with ART. Even so, the effect of such biomedical innovation on people's actual desires for traditional gestation may vary. For example, uterus transplantation (UTx) is a case where ART reinforces the importance of gestation as a central feature of reproduction, despite the fact that there are alternative ways to become a mother that do not involve gestation at all. UTx is a surgery aimed at allowing people without a uterus (women with absolute uterine factor infertility, mainly) to receive a uterus from a donor, so that they may experience a pregnancy in their own body. Interestingly, UTx might be perceived as more desirable than surrogacy precisely because the intended mother can experience embodied gestation.^34^ Discussions are currently taking place regarding the possibility for genetically XY recipients (e.g., transgender women or cisgender men) to have a pregnancy via UTx as well, even though clinical trials for this potential target group have not yet taken place.

Given that the possibilities enabled by ART have already sparked discussions about whose bodies should be able to gestate, who can become a parent, and so forth, it is in some sense unsurprising that those whose options for pregnancy are limited might deploy ART as a way to narrativise new and creative relationships to gestation. Gestational surrogacy already constitutes a unique example of a social arrangement in which the non‐gestating intended parent(s) are heavily invested in, and even anxious about, a pregnancy taking place in another person's body. Indeed, the close relationship between the gestational carrier and the non‐gestating intended parent(s) may be experienced as an attempt to nurture what might be referred to as an ‘alien’ pregnancy,^35^ which might include fears about miscarriage and the health of the child. In this way, we can see how even non‐gestating intended parents can become intimately involved—perhaps even overbearing—in a pregnancy that they do not experience in their own bodies, simply by enacting behaviours and attitudes that show proximate interests in an ‘alien’ pregnancy.

We postulate that a device like INVOcell might similarly be appropriated for related purposes—that is, as an opportunity to write oneself into the process of gestation, in case it is not possible for one to gestate according to the traditional understanding of the term. Based on the commonplace framings of INVOcell that we have already observed in the previous sections, it does not seem unreasonable to hypothesise that the device might serve an important *emblematic* purpose for the non‐gestating intended mother to feel involved in the process of gestation, especially against strongly bioheteronormative backgrounds of family‐making in which the expectation might have been for her to gestate. This possibility, then, makes it coherent for us to outline the following concept:

*Simulated gestation*; derivative, proximate, or symbolic participation in, or identification with, ‘gestation’ traditionally understood.

We have deliberately left this concept of simulated gestation open to interpretation, so as to accommodate the fact that people who cannot gestate in the traditional sense may still wish to play a role in the very same gestational process in more diverse ways. Indeed, even in heteronormative relationships, male partners may be doing this in concert with their gestating partners to a limited extent: by being proximate to the affective trajectory of their partner's pregnancy, men may come to feel that they are ‘expecting’,^36^ too, especially if they also held a desire to actually experience pregnancy like their partner.

In our INVOcell example, it seems that the ‘simulation’ is performed to the fullest extent, since the user would be utilising her own body to reinscribe and satisfy pre‐existing desires for traditional gestation. As we have mentioned in previous sections, the marketing of INVOcell is scientifically and morally suspect, and yet clearly effective at exploiting the bioessentialist motherhood ideals that might appeal to users. INVOcell claims to provide a more ‘natural’ way of experiencing motherhood, though one must question how its body‐independent functionality aligns with this claim. Given that INVOcell operates independently of the body, it is possible to place the device in an external environment at the fertilisation temperature, where it would still function effectively. The ability of INVOcell to enable fertilisation in a body‐independent manner parallels common IVF procedures in a laboratory dish. Yet, by marketing this process as ‘natural’ gestation, INVOcell endeavours to convince its users that the IVC device is preferable to IVF and actively encourages users to perform ‘gestation’ with the device.

As such, participation in gestation can be simulated—in potentially positive ways, but also in morally ambivalent ways—with the help of persons, technologies and other entities outside of the relevant individual. It appears, in short, a way for one to get around the logical gap between the desire to gestate and the impossibility to do so. In the INVOcell case, the logical impossibility to be negotiated is the fact that two lesbian aspiring mothers would both wish to gestate, even though it is not possible for two bodies to gestate the same foetus. INVOcell then becomes the representative device through which the non‐gestating party might try to narrativise their own body as causally necessary for the gestation to occur. Hence, the idea that placing this device in one's body is a type of ‘gestation’ *derives* from the hope and belief that it can compare with the kind of gestation that the intended gestator might experience; the act of having the device in one's body thereafter gains value due to its proximate placement with another person's pregnancy; and finally, the process is symbolic of one's desires to gestate and simultaneously identified *as* gestation.

In a similar manner, INVOcell could enable a scenario where the device is inserted into the body of a trans woman by surgery (who lacks a uterus), offering her the opportunity to experience a form of simulated gestation. In this case, the trans woman might wish to identify herself as a gestational mother, despite the absence of the traditional biological foundations of pregnancy. This scenario also strengthens the conventional understanding of motherhood, which has historically been tied to biological sex and the ability to carry and give birth to a child. While a trans woman would not undergo the typical physiological processes associated with pregnancy in a female‐bodied individual, the INVOcell device could be presented as a way to imitate nature by avoiding or bypassing the absence of the uterus to experience gestation in a symbolic way. Hence, INVOcell could develop a marketing narrative of inclusivity by allowing a trans woman to experience a supposedly pregnancy‐like process. The biomimetic practices would be anchored in the traditional ideas of motherhood that identify it with biology. In this imaginary, it is the mother's body that participates in a nurturing role and acts as the caring environment. Rather than alleviating the pressure that certain groups may feel for not fitting into gendered expectations, however, these discourses are potentially harmful insofar as they reinforce essentialist ideas of the gendered maternal experience. Furthermore, it does not only potentially cause emotional damage, as above, but also exposes users to unnecessary physical risks, for instance because of operations to insert and remove the device (given that INVOcell does not need the human body to function). However, qualitative research exploring the experiences of transgender people and simulated pregnancy would be very valuable in order to assess, in‐depth, the challenges that this technology poses for the group.

The fact that some people without access to traditional gestation might find the idea of simulated gestation alluring, however, does not necessarily commit us to any normative position about the desirability of gestation itself. That is, the actual practices of simulated gestation, such as the INVOcell protocol, should not necessarily be used to *support* the view that gestational motherhood is an overall positive phenomenon to reinforce through ART. Rather, the primary advantage of articulating a concept like simulated gestation is that it does not necessarily define gestation in a strict way, as has been done in the case of traditional gestation; thus, our concept can more easily recognise the fact that even ‘non‐gestating’ agents might, after all, find creative avenues to subjectively identify diverse reproductive experiences *as* gestational experiences. Thus, while in the case of INVOcell we might criticise how agents are reconstructing the symbolic meaning of ‘gestation’ through ART (e.g., by making biologically essentialist assumptions about the female body as an incubator), this does not change the fact that *simulating* gestation in these creative (if potentially objectionable) ways might be psychologically important to aspiring parents who cannot gestate the traditional way. Furthermore, our concept elucidates the strategies that the ART system deploys to maintain itself, namely, through the sale of new products and the construction of narratives about pregnancy and motherhood that make use of the anticipated desires and expectations of users. Our insights hold irrespective of whether the practice or phenomenon of simulated gestation *should* ultimately be encouraged. Moreover, it seems likely that novel understandings of gestation will become increasingly more relevant, given the changes that have already taken place in reproductive processes due to ART. For instance, development of artificial placenta technology for partial or even full gestation outside of the human body will no doubt be the next realm in which philosophical renegotiations about gestation will take place. If gestation can be done entirely outside of a human body, people may nevertheless look for ways to personally relate to *ecto*gestation^37^￼. Such possibilities make the concept of simulated gestation highly relevant as an explanatory framework that appreciates the diverse psychoaffective desires of aspiring gestators in parallel with ongoing ART development.

Our view is that articulating this concept of simulated gestation fulfils a descriptive, as well as normative, purpose. It fulfils a descriptive purpose by being able to offer an explanation of why users of INVOcell might value, and are prone to perform, traditional ‘gestation’ even in cases where it is not, strictly speaking, taking place. Moreover, simulated gestation fulfils a normative purpose by anticipating, and giving importance to, the ways in which people's ideas about gestation are evolving over time and in response to emerging reproductive technologies.

## 4 CONCLUSION

In this article, we have presented the effective marketing strategy of INVOcell as having morally ambivalent implications for users' conceptualisations about gestation. While marketed as a more natural and intimate alternative to IVF, INVOcell's promotion relies heavily on fallacious naturalistic and essentialist narratives which emphasise the female body's role in gestation. This marketing strategy can be problematic, as it perpetuates the notion that gestation is a necessary component of motherhood, thereby marginalising other routes into motherthood like adoption or surrogacy. Furthermore, appealing to potential service users with ethical, religious and cultural objections to IVF by promoting the ‘natural’ in vivo process of INVOcell risks reinforcing harmful stereotypes and misleading potential users about the true function of the technology. While technologies like INVOcell provide new opportunities for reproductive decision‐making, they also necessitate ongoing scrutiny to ensure that they do not reinforce exclusionary or oppressive norms. Users should at the very least be aware and informed that they are not necessarily playing an active causal role in the conception process or the embryo culture process. This may prevent the potential for emotional distress resulting from the failure of the process or from the inability to obtain an adequate number of viable embryos for implantation.

To add nuance to our critical analysis, we introduced the concept of simulated gestation as an explanatory lens through which the purported value of INVOcell could be further investigated. The concept of simulated gestation reflects a broader trend in ART where biological and symbolic participation in gestation is defined not only by the science of the reproductive process, but also negotiated by its users to fulfil psychological and social desires for parenthood. At the individual level, simulated gestation may offer inclusivity and psychological benefits by allowing both partners to feel involved in the reproductive process. However, in the collective dimension, it may reinforce essentialist ideas about the creation of kinship and familial bonds by overestimating the significance of biological over social ties. Either way, we hope that our critical evaluation of INVOcell demonstrates an ongoing need to evaluate how ART shapes and reshapes societal perceptions of gestation and motherhood.

## Footnotes

^1^
Jellerette‐Nolan, T., Cooper, A. R., Doody, K. J., Nichols, J. E., Park, J. K., Poe‐Zeigler, R. L., Khair, A. K., Stong, L. M., Paulson, M. D., & Daftary, G. S. (2020). Real‐world experience with intravaginal culture using INVOCELL: An alternative model for infertility treatment. *F&S Reports*, *2*(1), 9–15; Aguiar, L. de F., Cunha, G. dos S. P. da, Cordido, K. A. A. T., Coelho, F. A. C., & Ortiga‐Carvalho, T. M. (2023). Randomized prospective study comparing conventional In Vitro Fertilisation technique to Intravaginal Culture with the INVOCELL^TM^ device for 3 and 5 days. *JBRA Assisted Reproduction*, *27*(3), 360–366; Doody, K. J., Broome, E. J., & Doody, K. M. (2016). Comparing blastocyst quality and live birth rates of intravaginal culture using INVOcell^TM^ to traditional in vitro incubation in a randomized open‐label prospective controlled trial. *Journal of Assisted Reproduction and Genetics*, *33*(4), 495–500.

^2^
As we elaborate in the following paragraphs, it is worth noting that the recipient of an INVOcell device is not necessarily the same person carrying the pregnancy from implantation to delivery.

^3^

https://www.invocell.com

^4^
Babcock Gilbert, S., & Polotsky, A.J. (2019). Vaginal culture for IVF allows two mothers to carry the same pregnancy: Is more always better? *Case Reports in Women's Health*, *21*, e00099.

^5^

https://www.livescience.com/63975-two-mothers-carry-baby-invocell.html

^6^

https://euroweeklynews.com/2023/10/31/europes-first-pregnancy-shared-by-two-women/

^7^

https://www.oed.com/

^8^
Fishel, S. (2018). First in vitro fertilisation baby—This is how it happened. *Fertility and Sterility*, *110*(1), 5–11; Johnson, M. H. (2019). A short history of in vitro fertilisation (IVF). *The International Journal of Developmental Biology, 63*(3‐4–5), 83–92; Johnson, M. (2019). Human in vitro fertilisation and developmental biology: a mutually influential history. *Development (Cambridge, England), 146*(17).

^9^
Leeton, J. (2004). The early history of IVF in Australia and its contribution to the world (1970‐1990). *The Australian & New Zealand Journal of Obstetrics & Gynaecology*, *44*(6), 495–501.

^10^
Franklin, S. (2013). *Biological relatives: IVF, stem cells, and the future of kinship* (p. 364). Duke University Press.

^11^
Woolgar, S. (1990). Configuring the user: The case of usability trials. *The Sociological Review*, *38*(1_suppl), 58–99.

^12^
Hurlbut, J. B. (2015). Remembering the future: Science, law and the legacy of Asilomar. In S. Jasanoff, & S.‐H. Kim (Eds.), *Dreamscapes of modernity: Sociotechnical imaginaries and the fabrication of power* (pp. 126–151). University of Chicago Press.

^13^
Geels, F. W. (2007). Transformations of large technical systems. *Science, Technology, & Human Values*, *32*(2), 123–149; Smith, M. R. (1994). Technological determinism in American culture. In *Does technology drive history? The dilemma of technological determinism* (pp. 1–35). MIT Press.

^14^
Mamo, L. (2007). Queering reproduction: Achieving pregnancy in the Age of Technoscience. Duke University Press.

^15^
Fernández‐Jimeno, N. (2024). How sociotechnical systems adapt to change. *Science & Technology Studies*, 1–19.

^16^

https://www.invocell.com

^17^
Mastroyannis, C. (1993). Gamete intrafallopian transfer: ethical considerations, historical development of the procedure, and comparison with other advanced reproductive technologies. *Fertility and Sterility*, *60*(3), 389–402.

^18^

https://www.diariodemallorca.es/mallorca/2023/03/05/mallorca-primer-embarazo-pais-pareja-84165301.html

^19^

https://www.invobioscience.com/patients/

^20^
Arp, R., Barbone, S., & Mike, M. B. (2018). Bad Arguments: 100 of the Most Important Fallacies in Western Philosophy. John Wiley & Sons.

^21^
Hoffmann, L., Hilger, N., & Banse, R. (2023). The mindset of birth predicts birth outcomes: Evidence from a prospective longitudinal study. *European Journal of Social Psychology*, *53*(5), 857–871.

^22^
Smajdor, A., Cutas, D., & Takala, T. (2018). Artificial gametes, the unnatural and the artefactual. *Journal of Medical Ethics*, *44*(6), 404–408.

^23^

https://www.juaneda.es/blog/337/nace-en-juaneda-hospitales-el-primer-bebe-de-europa-gestado-conjuntamente-por-una-pareja-de-mujeres-gracias-al-metodo-invocell

^24^

https://www.invobioscience.com/patients/

^25^
Murphy, T. F., & Parks, J. A. (2020). Gestation as mothering. *Bioethics*, *34*(9), 960–968.

^26^
Kingma, E. (2019). Were you a part of your mother? *Mind*, *128*(511), 609–646.

^27^

https://aeon.co/essays/is-the-mother-a-container-for-the-foetus-or-is-it-part-of-her

^28^
Cutas, D. (2023). Nothing if not family? Genetic ties beyond the parent/child dyad. *Bioethics*, *37*(8), 763–770.

^29^
Robertson, J. A. (1996). Assisted reproductive technology and the family. *Hastings Law Journal*, *47*(4), 911.

^30^

https://www.juaneda.es/blog/337/nace-en-juaneda-hospitales-el-primer-bebe-de-europa-gestado-conjuntamente-por-una-pareja-de-mujeres-gracias-al-metodo-invocell

^31^
Brandão, P., de Pinho, A., Ceschin, N., Sousa‐Santos, R., Reis‐Soares, S., & Bellver, J. (2022). ROPA—Lesbian shared in vitro fertilisation—Ethical aspects. *European Journal of Obstetrics and Gynecology and Reproductive Biology*, *272*, 230–233.

^32^
Reproducing the Future: Anthropology, Kinship, and the New Reproductive Technologies, Marilyn Strathern. 1992. Routledge, Chapman & Hall, New York, NY. 224 pages. ISBN: 0‐415‐90555‐9 (hc); 0‐415‐90556‐7 (pb). (1994). *Bulletin of Science, Technology & Society*, *14*(1), 62–62.

^33^

https://www.europarl.europa.eu/RegData/etudes/BRIE/2023/754562/EPRS_BRI(2023)754562_EN.pdf

^34^
Robertson, J. A. (2016). Other women's wombs: uterus transplants and gestational surrogacy. *Journal of Law and the Biosciences*, *3*(1), 68.

^35^
Majumdar, A. (2014). Nurturing an alien pregnancy: Surrogate mothers, intended parents and disembodied relationships. *Indian Journal of Gender Studies*, *21*(2), 199–224.

^36^
Jonathan, A. (2022). *‘Husbands are pregnant, too’: Caring masculinities in pregnancy books for men*. https://doi.org/10.1177/10608265221122799

^37^
Lee, J. Y. (2022). Framing gestation: Assistance, delegation, and beyond. *Journal of Medical Ethics*, *48*(7), 448–449.

